# Supplementary material for: The HASTER lncRNA promoter is a cis-acting transcriptional stabilizer of HNF1A
Source: Nat Cell Biol. 2022 Oct 6;24(10):1528–40. doi: 10.1038/s41556-022-00996-8 (PMC9586874; doi:10.1038/s41556-022-00996-8)
Supplement: Supplementary file 1 — Reporting Summary [file 41556_2022_996_MOESM1_ESM.pdf]

Corresponding author(s): Jorge Ferrer

Last updated by author(s): 2022/04/17

Reporting Summary

Nature Research wishes to improve the reproducibility of the work that we publish. This form provides structure for consistency and transparency in reporting. For further information on Nature Research policies, see our Editorial Policies and the Editorial Policy Checklist.

Statistics

For all statistical analyses, confirm that the following items are present in the figure legend, table legend, main text, or Methods section.

n/a	Confirmed	
☐	☒	The exact sample size (*n*) for each experimental group/condition, given as a discrete number and unit of measurement
☐	☒	A statement on whether measurements were taken from distinct samples or whether the same sample was measured repeatedly
☐	☒	The statistical test(s) used AND whether they are one- or two-sided<br>*Only common tests should be described solely by name; describe more complex techniques in the Methods section.*
☒	☐	A description of all covariates tested
☐	☒	A description of any assumptions or corrections, such as tests of normality and adjustment for multiple comparisons
☐	☒	A full description of the statistical parameters including central tendency (e.g. means) or other basic estimates (e.g. regression coefficient) AND variation (e.g. standard deviation) or associated estimates of uncertainty (e.g. confidence intervals)
☐	☒	For null hypothesis testing, the test statistic (e.g. *F*, *t*, *r*) with confidence intervals, effect sizes, degrees of freedom and *P* value noted<br>*Give P values as exact values whenever suitable.*
☒	☐	For Bayesian analysis, information on the choice of priors and Markov chain Monte Carlo settings
☒	☐	For hierarchical and complex designs, identification of the appropriate level for tests and full reporting of outcomes
☒	☐	Estimates of effect sizes (e.g. Cohen's *d*, Pearson's *r*), indicating how they were calculated

Our web collection on statistics for biologists contains articles on many of the points above.

Software and code

Policy information about availability of computer code

Data collection

sra-tools (v.2.9.1_1)
loomR (v.0.2.0.1)
loompy (v.2.0.16)

Data analysis

bedGraphToBigWig (v4)
Bowtie2 (v.2.3.5)
Samtools (v.1.7)
BedTools (v.2.27.1)
MACS2 (v.2.1.1)
DiffBind (v.2.8.0)
deepTools (v.3.0.2)
pybedtools (v.0.8.0)
Homer (v.3.12)
Picard (v.2.6.0)
GSEAPreranked (v.6.0, GenePattern)
CellRanger (v.3.0.2)
Seurat (v.3.0.1)
scVI-tools (v.0.11.0)
python (v.3.7.3)
numpy (v.1.15.4)
pandas (v.0.25.0)
matplotlib (v.3.1.0)
seaborn (v.0.9.0)
scipy (v.1.1.0)

STAR (v.2.3.0)
Salmon (v.0.11)
r-base (v.3.6.1)
DESeq2 (v.1.24.0)
StringTie (v.2.0)
gffcompare (v.0.10.1)
umi4cPackage (v 0.0.0.9000)
ea-utils (v.1.3.1)

For manuscripts utilizing custom algorithms or software that are central to the research but not yet described in published literature, software must be made available to editors and reviewers. We strongly encourage code deposition in a community repository (e.g. GitHub). See the Nature Research guidelines for submitting code & software for further information.

Data

Policy information about availability of data

All manuscripts must include a data availability statement. This statement should provide the following information, where applicable:

- Accession codes, unique identifiers, or web links for publicly available datasets
- A list of figures that have associated raw data
- A description of any restrictions on data availability

Raw sequence reads from RNA-seq, scRNA-seq, UMI-4C and ChIP-seq are available from Arrayexpress, under accession number (E-MTAB-11463, E-MTAB-11471, E-MTAB-11472, E-MTAB-11473, E-MTAB-11474, E-MTAB-11475 and E-MTAB-11477).
Mouse liver ChIP-seq for CTCF was from GSE29184; RAD21 from GSE102997; FOXA2, CEBPB and HNF4A from GSE57559; PPARA from GSE108689; RXR from GSE35262; GATA4 from GSE49132. Mouse ATAC-seq was from SRP167062.
Liver RNA-seq for human is from SRX218942, chicken from SRX2704301, X. tropicalis from SRX2704321 and zebrafish from E-MTAB-8959.
RNA-seq of hPSC differentiated to β cells were obtained from GSE140500.
Mouse kidney (SRX2370375) and small intestine (SRX2370402) RNA-seq reads were obtained from the Mouse ENCODE project
GTex data was downloaded from https://gtexportal.org/home/
GRCm38 Bowtie2 index was downloaded from https://genome-idx.s3.amazonaws.com/bt/mm10.zip
GRCh37 human genome was downloaded from GENCODE https://www.gencodegenes.org/human/release_19.html
GRCm38 mouse genome was downloaded from https://www.gencodegenes.org/mouse/release_M10.html
GRCz11 (dnaRer11) zebrafish genome was downloaded from https://www.ncbi.nlm.nih.gov/assembly/GCF_000002035.6/
galGal5 chicken genome and XenTro9 Xenopus genome were downloaded from ENSEMBL (www.ensembl.org)
Processed data files are provided as Supplementary Data Sets and deposited at https://www.crg.eu/en/programmes-groups/ferrer-lab#datasets (pending at submission).

Field-specific reporting

Please select the one below that is the best fit for your research. If you are not sure, read the appropriate sections before making your selection.

☒ Life sciences ☐ Behavioural & social sciences ☐ Ecological, evolutionary & environmental sciences

For a reference copy of the document with all sections, see nature.com/documents/nr-reporting-summary-flat.pdf

Life sciences study design

All studies must disclose on these points even when the disclosure is negative.

Sample size	For in vivo experiments, sample sizes were defined based on studies with similar experimental design and expected outcome. For glucose tolerance curves the numbers used are expected to yield statistically significant differences for >10% glucose tolerance effects of single gene defects bred on an inbred background. For in vitro studies, we ensured that any result is not only robust to technical replication of the read-out assay, but also to different attempts to perform the genetic modification. For CRISPR deletions, CRISPRi road block and CRISPR activation on cell lines, all experiments were performed using at least 2 different sgRNAs, or 2 different pair of sgRNA for deletions. Knock-down experiments were performed with 2 different LNA Gapmers. Likewise we used at least 2 independent mutant and control clones for hPSC mutations.
Data exclusions	No data were excluded.
Replication	In addition to using independent modifications as outlined above, each independent modification was studies with replicate experiments, as detailed for each result. Immunofluorescence stainings were performed on tissues from 2 or 3 mice for each genotype and age. Western blot and RNA-smFISH experiments were all replicated 2 to 3 times. All attempt of replication reported were successful.
Randomization	Not relevant for this study. Samples were allocated to a group based on their genotypes.
Blinding	While no proper blinding were performed, no particular attention was given to the genotypes during samples collection or measurements.

Reporting for specific materials, systems and methods

We require information from authors about some types of materials, experimental systems and methods used in many studies. Here, indicate whether each material, system or method listed is relevant to your study. If you are not sure if a list item applies to your research, read the appropriate section before selecting a response.

Materials & experimental systems

n/a	Involved in the study
☐	☒ Antibodies
☐	☒ Eukaryotic cell lines
☒	☐ Palaeontology and archaeology
☐	☒ Animals and other organisms
☒	☐ Human research participants
☒	☐ Clinical data
☒	☐ Dual use research of concern

Methods

n/a	Involved in the study
☐	☒ ChIP-seq
☒	☐ Flow cytometry
☒	☐ MRI-based neuroimaging

Antibodies

Antibodies used

Rabbit anti-β-Tubulin (#2146, Cell Signaling Technology)
Rabbit anti-HNF1A (D7Z2Q, #89670, Cell Signaling Technology)
Guinea pig anti-insulin (A0564, Dako, discontinued)
Guinea pig anti-glucagon (polyclonal, 4030-01F, Millipore, discontinued)
Rat anti-Cytokeratin 19 (Hybridoma Bank, TROMA III-c)
Goat anti-PDX1 (AF2419, R&D Systems)
Mouse anti-Nkx6.1 (F55A10, Hybridoma Bank)
Rabbit anti-H3K27ac (Abcam, ab4729)
Rabbit monoclonal anti-H3K4me3 (Merk, 05-745R, clone 15-10C-E4)
Rabbit anti-H3K4me1 (Abcam, ab8895)
Donkey anti rabbit Alexa Fluor 488 (711-545-152, Jackson Immunoresearch, 1/800)
Donkey anti rabbit Alexa Cy3 (711-166-152, Jackson Immunoresearch, 1/400)
Donkey anti guinea pig Alexa Fluor 488 (706-545-148, Jackson Immunoresearch, 1/800)
Donkey anti guinea pig Cy5 (706-175-148, Jackson Immunoresearch, 1/400)
Donkey anti goat Alexa Fluor 488 (705-545-147, Jackson Immunoresearch, 1/800)
Donkey anti rat Cy3 (712-165-153, Jackson Immunoresearch, 1/400)
Donkey anti mouse Cy5 (715-175-151, Jackson Immunoresearch, 1/400)
Goat Anti-Rabbit IgG H&L (HRP) (ab97051, Abcam, 1/2000)

Validation

The anti-β-Tubulin has been tested for Western blot by the provider using various cell lines extracts. This antibody is widely used (676 citations).
The anti-HNF1A was tested on section from acinar-specific Hnf1a KO mice (Kalisz et al, EMBO J, 2020)
The anti-insulin, anti-glucagon and anti-Cytokeratin 19 antibodies are widely used in the field (http://www.informatics.jax.org/antibody/key/1494).
The anti-PDX1 was validated by by Western blot and immunohistochemistry by manufacturer (https://www.rndsystems.com/products/human-pdx-1-ipf1-antibody_af2419)
Anti-Nkx6.1 (Generation and characterization of monoclonal antibodies against the transcription factor Nkx6.1, Jorgensen MC, The journal of histochemistry and cytochemistry : official journal of the Histochemistry Society 54.5 (2006 May): 567-74.
The anti-H3K4me3 was validated by dotblot for the ENCODE project (https://genome.ucsc.edu/ENCODE/validation/antibodies/human_H3K27ac_validation_Bernstein.pdf)
The anti-H3K4me3 was validated by ChIP-seq by the manufacturer "The highest 25% of peaks identified in the 05-745R and 07-473 datasets showed 99% overlap with peaks identified in the ENCODE H3K4me3 BROAD Histone track for HeLa S3." https://www.merckmillipore.com/GB/en/product/Anti-trimethyl-Histone-H3-Lys4-Antibody-clone-15-10C-E4-rabbit-monoclonal,MM_NF-05-745R?ReferrerURL=https%3A%2F%2Fwww.google.com%2F&bd=1
The anti-H3K4me1 was was validated by dotblot for the ENCODE project (https://genome.ucsc.edu/ENCODE/validation/antibodies/mouse_H3K4me1_ab8895_validation_Ren.pdf)

Eukaryotic cell lines

Policy information about cell lines

Cell line source(s)

EndoC-βH3 cells were generated and provided by Philippe Ravassard, a co-author of this study (Benazra et al., Mol Metab., 2015).
MIN6 cells (Miyazaki, et al., Endocrinology, 1990) were provided by Rosa Gasa (IDIBAPS, Barcelona, Spain)
H9 cells were provided by WiCell.
293FT were purchased from from Invitrogen.

Authentication

Cell lines were authenticated by morphological inspection and qPCR measurement of cell-type-specific markers.

Mycoplasma contamination

EndoC-βH3 and H9 cells were tested and were negative for Mycoplasma. MIN6 and 293FT were not tested.

Commonly misidentified lines
(See ICLAC register)

None of the commonly misidentified lines were used.

Animals and other organisms

Policy information about studies involving animals; ARRIVE guidelines recommended for reporting animal research

Laboratory animals	When not specified otherwise, 8- to 12-weeks old males were used for sample collection and glucose stimulated insulin secretion. The Haster Stop and Haster floxed alleles were generated for this study in C57BL/6 background. The Haster null allele was obtained by breeding the Haster floxed allele with the Tg(EIIa-cre) mice. Cell type specific deletions were performed using the Alb-Cre (Tg(Alb1-cre)1Khk), Pdx1-Cre (Tg(Pdx1-Cre)6Tuv), Ins1-Cre (Ins1tm1.1(cre)Thor) and Pdx1CreERTM (Tg(Pdx1-Cre/Esr1*)1Mga). For allele specific experiments, Haster +/Stop or Haster +/- mice were bred with PWK/PhJ mice and the F1 was analyzed.
Wild animals	This study did not involve wild animals.
Field-collected samples	This study did not involve samples collected from the field.
Ethics oversight	Animal experimentation was carried out in compliance with the EU Directive 86/609/EEC and Recommendation 2007/526/EC regarding the protection of animals used for experimental and other scientific purposes, enacted under Spanish law 1201/2005, and all experiments were approved by the Institutional Animal Care Committees of the University of Barcelona and Parc de Recerca Biomedica de Barcelona.

Note that full information on the approval of the study protocol must also be provided in the manuscript.

ChIP-seq

Data deposition

☒ Confirm that both raw and final processed data have been deposited in a public database such as GEO.

☒ Confirm that you have deposited or provided access to graph files (e.g. BED files) for the called peaks.

Data access links<br>*May remain private before publication.*	Processed data files are provided as Supplementary Data Sets and deposited at https://www.crg.eu/en/programmes-groups/ferrer-lab#datasets (pending at submission).
Files in database submission	Access from Arrayexpress pending at the time of submission
Genome browser session<br>(e.g. UCSC)	no longer applicable

Methodology

Replicates

ChIP-seq were performed on 3 KO and 3 control mice for each antibody used. Between 84% to 98% of peaks from the replicate with the lowest number of peaks called were present in the other replicates.

Sequencing depth

Sample	Antibody	Read length	Total number of reads	Uniquely aligned reads (after removing duplicates)
Haster_LKO_1	HNF1A	50 SE	36,577,365	26,191,763
Haster_LKO_2	HNF1A	50 SE	36,412,249	26,168,607
Haster_LKO_3	HNF1A	50 SE	34,102,415	24,137,402
Control_1	HNF1A	50 SE	29,209,244	20,679,931
Control_2	HNF1A	50 SE	40,716,830	29,132,320
Control_3	HNF1A	50 SE	30,681,737	17,823,581
Haster_LKO_1	H3K4me3	50 SE	40,778,667	25,266,443
Haster_LKO_2	H3K4me3	50 SE	40,134,454	24,487,328
Haster_LKO_3	H3K4me3	50 SE	41,345,041	24,344,704
Control_1	H3K4me3	50 SE	23,870,270	16,172,657
Control_2	H3K4me3	50 SE	49,820,846	29,578,339
Control_3	H3K4me3	50 SE	30,669,001	17,572,781
Haster_LKO_1	H3K27ac	50 SE	40,318,283	33,806,790
Haster_LKO_2	H3K27ac	50 SE	51,441,794	42,494,143
Haster_LKO_3	H3K27ac	50 SE	48,839,781	40,112,808
Control_1	H3K27ac	50 SE	29,620,302	24,993,138
Control_2	H3K27ac	50 SE	51,638,898	42,321,953
Control_3	H3K27ac	50 SE	30,567,140	26,055,207

Antibodies

Rabbit anti-HNF1A (D7Z2Q, Cell Signaling Technology)
Rabbit anti-H3K27ac antibody (Abcam, ab4729)
Rabbit monoclonal anti-H3K4me3 (Merk, 05-745R, clone 15-10C-E4)

Peak calling parameters

```
BOWTIE_INDEX=bowtie2-index/mm10/mm10
BLACKLISTED_REGIONS=mm10.blacklist.bed
#Aligning reads with Bowtie2
bowtie2 -p 16 --no-unal -x $BOWTIE_INDEX -U $FASTQ_FILE_PATH/$FASTQ_FILE -S $OUTPUT_DIR/${sample_name}_aligned.sam

#Converting sam file to bam format, filter for multimappings
samtools view -q 30 -bh -o $OUTPUT_DIR/${sample_name}_aligned.bam $OUTPUT_DIR/${sample_name}_aligned.sam
```

```
echo "Sort bam"
samtools sort -@ 16 -T $TMPDIR/${sample_name}_aligned.tmp.bam -o $OUTPUT_DIR/${sample_name}_sorted.bam $OUTPUT_DIR/
${sample_name}_aligned.bam

# Remove duplicates
picard MarkDuplicates \
    I=$OUTPUT_DIR/${sample_name}_sorted.bam \
    O=$OUTPUT_DIR/${sample_name}_nodup.bam \
    M=$OUTPUT_DIR/${sample_name}_nodup.log \
    REMOVE_DUPLICATES=true

#Filter out blacklisted regions
intersectBed -abam  $OUTPUT_DIR/${sample_name}_nodup.bam -b $BLACKLISTED_REGIONS -v > $OUTPUT_DIR/
${sample_name}_clean.bam

# Indexing bam file
samtools index $OUTPUT_DIR/${sample_name}_clean.bam

#Peak calling for transcription factor ChIP
macs2 callpeak -t $OUTPUT_DIR/${sample_name}_clean.bam -c $INPUT_CHIP -f BAM -g mm -n ${MACS2_OUTPUT} --keep-dup all -B
-q 0.05 --outdir $OUTPUT_DIR

#Peak calling for transcription factor ChIP
macs2 callpeak -t $OUTPUT_DIR/${sample_name}_clean.bam -c $INPUT_CHIP -f BAM -g mm -n ${MACS2_OUTPUT} --keep-dup all -B
-q 0.05 --broad --outdir $OUTPUT_DIR
```

Data quality

Peaks called at in 2 replicates were merged and used for differential binding and subsequent analysis.

Sample	Antibody	Percentage of peaks called at 5% FDR with >5-fold enrichment
Haster_LKO_1	HNF1A	51.7%
Haster_LKO_2	HNF1A	54.7%
Haster_LKO_3	HNF1A	60.6%
Control_1	HNF1A	69.4%
Control_2	HNF1A	48.9%
Control_3	HNF1A	75.6%
Haster_LKO_1	H3K4me3	46.8%
Haster_LKO_2	H3K4me3	50.4%
Haster_LKO_3	H3K4me3	52.2%
Control_1	H3K4me3	54.4%
Control_2	H3K4me3	46.6%
Control_3	H3K4me3	58.7%

Software

Reads were aligned using Bowtie2 (v.2.3.5)
Duplicates were removed using Picard (v.2.6.0)
Peaks were called using MACS2 (v.2.1.1)
Intersections were performed using BedTools (v.2.27.1)
Differential binding sites were determined using DiffBind (v.2.8.0)
Read coverages were computed using deepTools (v.3.0.2)
Motif analysis was performed using Homer (v.3.12)
