# Supplementary material for: The HASTER lncRNA promoter is a cis-acting transcriptional stabilizer of HNF1A
Source: Nat Cell Biol. 2022 Oct 6;24(10):1528–40. doi: 10.1038/s41556-022-00996-8 (PMC9586874; doi:10.1038/s41556-022-00996-8)

Experiment 1

Long exposure

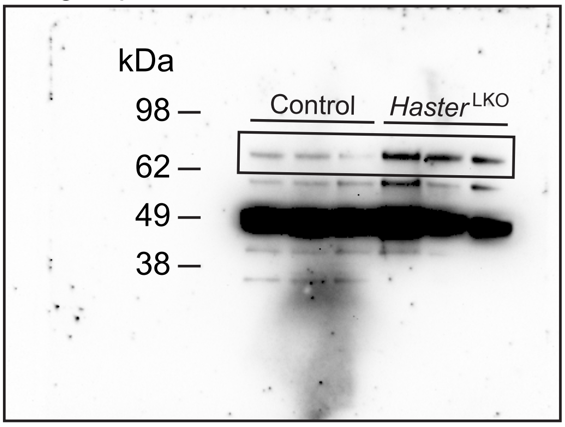

Selected regions are represented in Figure 2f

HNF1A (67 kDa)

β-Tubulin (50 kDA)

Short exposure

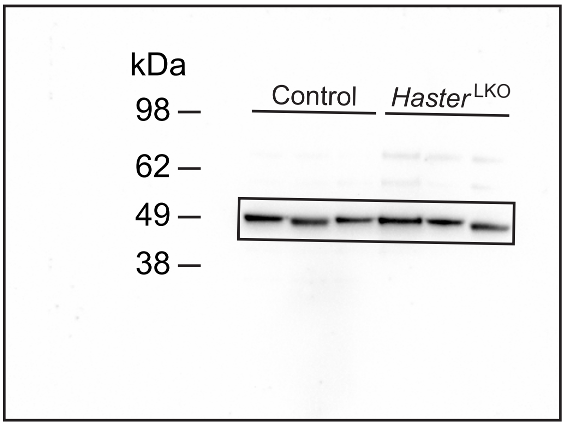

HNF1A (67 kDa)

β-Tubulin (50 kDa)

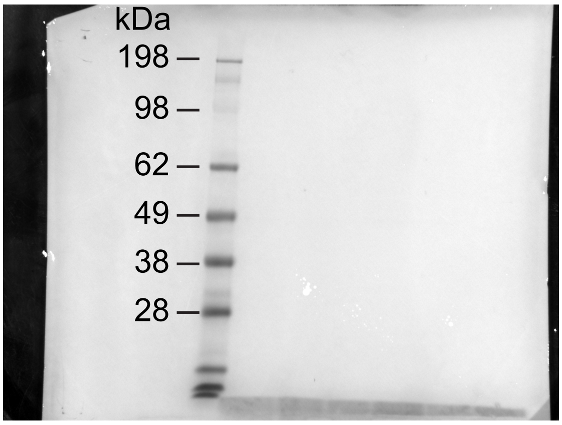

Experiment 2

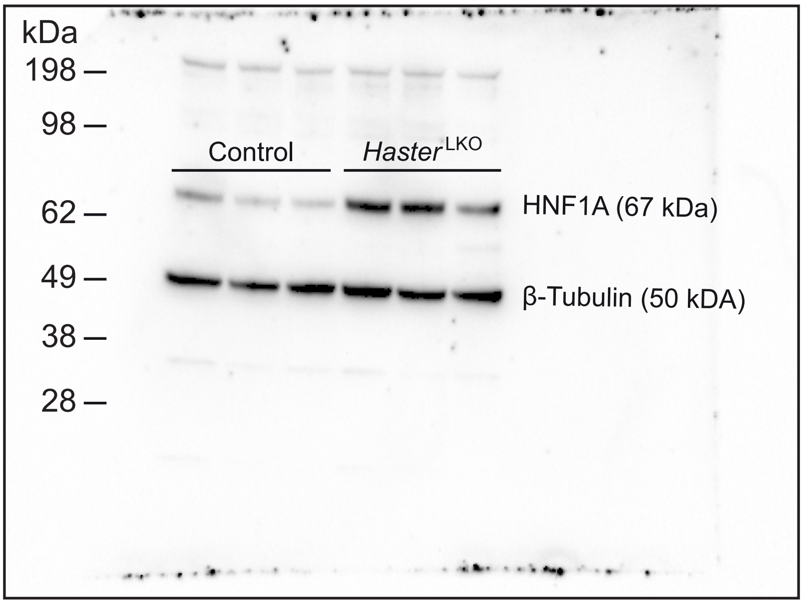

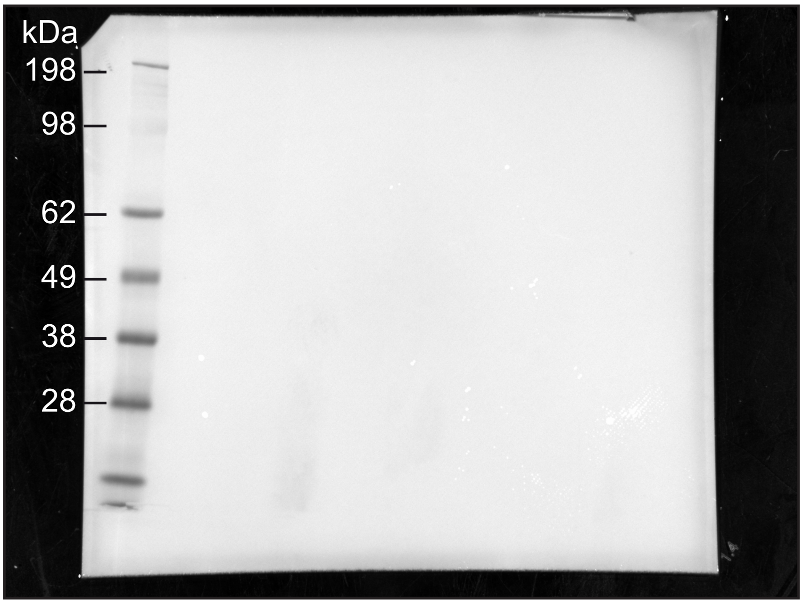

Supplement: Source Data Fig. 2 — Unprocessed western blots. [file 41556_2022_996_MOESM5_ESM.pdf]
